# Secukinumab for Treatment of Plaque Psoriasis in Real-World Clinical Practice in Spain: A Literature Review

**DOI:** 10.3390/jcm14020478

**Published:** 2025-01-13

**Authors:** Esteban Daudén, José María Ortiz-Salvador, Jaime Notario, Lluís Puig, Jorge Santos-Juanes, Enrique Herrera-Acosta, Lara Gómez-Labrador, Ricardo Ruiz-Villaverde

**Affiliations:** 1 Dermatology Department, Hospital Universitario de la Princesa, Instituto de Investigación Sanitaria de La Princesa (IIS-IP), 28006 Madrid, Spain; estebandauden@gmail.com; 2 Dermatology Department, Hospital General Universitario de Valencia, 46014 Valencia, Spain; josema.ortiz.salvador@gmail.com; 3 Dermatology Department, Hospital Universitario de Bellvitge, 08907 Barcelona, Spain; jnotario@bellvitgehospital.cat; 4 Dermatology Department, Hospital de la Santa Creu i Sant Pau, 08025 Barcelona, Spain; lpuig@santpau.cat; 5 Dermatology Department, Hospital Universitario Central de Asturias, 33011 Oviedo, Spain; jorgesantosjuanes@gmail.com; 6 Dermatology Department, Hospital Clínico Virgen de la Victoria, 29010 Málaga, Spain; enrique.herrera.acosta.sspa@juntadeandalucia.es; 7 Novartis Farmacéutica S.A., 08013 Barcelona, Spain; lara.gomez_labrador@novartis.com; 8 Dermatology Department, Hospital Universitario San Cecilio, Instituto Biosanitario de Granada, Ibs, 18007 Granada, Spain

**Keywords:** psoriasis, real-world evidence, secukinumab, Spain

## Abstract

**Background/Objectives**: Secukinumab was shown to be effective in treating moderate-to-severe plaque psoriasis in adults and pediatric patients ≥6 years. **Methods**: A literature review was conducted to identify studies published in the preceding 5 years assessing the effectiveness and/or survival (safety in the second instance) associated with secukinumab treatment for moderate-to-severe plaque psoriasis with/without psoriatic arthritis (PsA) in real-world clinical practice in Spain. **Results**: 11 references were included, corresponding to seven studies (six retrospective and one prospective) (n = 1050). Baseline characteristics were mean age 46.5–53.0 years; 28.7–55.0% women; 30.0–53.1% patients with PsA; 27.9–47.4% naïve to biologic treatments; mean Psoriasis Area and Severity Index (PASI) score 12.5 (standard deviation [SD]: 6.9)–18.1 (SD: 10.3). PASI 90 response rates were 54–65% at Week 24 and 46–63% at Week 52; 43–47% of patients showed PASI ≤ 1 at Week 12 and 47–56% at Week 52. Treatment persistence at Week 52 was 72–89%, being 74.5% in the larger cohort series (n = 384) at 2 years. Adverse-event-related treatment discontinuation was rare. **Conclusions**: Secukinumab demonstrated long-term safety and efficacy in treating adult patients with moderate-to-severe plaque psoriasis in actual clinical practice, with survival rates of up to two years and consistent efficacy in Spain.

## 1. Introduction

Psoriasis is a chronic, recurrent inflammatory skin disease that affects approximately 1.5–3% of the general population in Europe [1]. In recent years, severe forms of psoriasis have been definitively associated with a range of comorbidities that significantly impact cardiovascular risk, establishing psoriasis as a systemic disease with predominantly cutaneous manifestations. Furthermore, severe psoriasis is now recognized as a condition that substantially impairs patients’ quality of life across physical, emotional, sexual, and economic domains. Diagnosis is primarily clinical, with histological confirmation rarely necessary. Plaque psoriasis, also known as psoriasis vulgaris, is the most common form, accounting for 85–90% of cases. It is characterized by well-demarcated, elevated erythematous plaques with scaling, typically distributed symmetrically on the extensor surfaces of the limbs, the scalp, and, to a lesser extent, the palms and soles. Up to 30% of patients may develop inflammatory joint disease, known as psoriatic arthritis [2,3].

Secukinumab, a fully human monoclonal antibody (IgG1) targeting interleukin-17A (IL-17A), a pro-inflammatory cytokine central to the pathogenesis of systemic inflammatory diseases such as psoriasis, represents a significant advancement in therapy [3]. Secukinumab is indicated for the treatment of moderate-to-severe plaque psoriasis in adults who are candidates for systemic therapy. The recommended dose is 300 mg administered subcutaneously (two injections of 150 mg) at weeks 0, 1, 2, and 3, followed by monthly maintenance doses starting at week 4. Treatment discontinuation is advised in cases of inadequate response at week 16.

The efficacy of secukinumab [4,5,6,7,8,9,10] has been demonstrated in a total of 10 phase 2 and phase 3 clinical trials involving 3430 patients with moderate-to-severe plaque psoriasis. Four pivotal studies evaluated the efficacy of secukinumab in inducing remission within 12 weeks of treatment initiation and provided maintenance data through week 52. Additional phase 3 trials assessed various maintenance regimens. Approximately 2000 patients continue in open-label extension studies, with follow-up durations ranging from 1 to 5 years. These studies evaluated two secukinumab doses (150 mg and 300 mg subcutaneously), demonstrating superiority over placebo in achieving a ≥75% improvement in baseline Psoriasis Area and Severity Index (PASI 75) and an Investigator’s Global Assessment (IGA mod 2011) score of 0 or 1 by week 12. In study A2303, an additional arm compared secukinumab to etanercept, confirming secukinumab’s non-inferiority and subsequent superiority at week 12 and in maintaining efficacy through week 52.

These trials enrolled adult patients with moderate-to-severe plaque psoriasis eligible for systemic therapy due to inadequate control with topical treatments, phototherapy, or prior systemic therapies [4,5,6,7,8,9,10]. Baseline PASI scores ranged from 20.1 to 23.7, and baseline body surface area (BSA) involvement ranged from 27.4% to 34.4%. Participants included both systemic therapy-naïve patients and those previously treated with systemic or biologic agents. Over half of the patients (55.5% to 67%) had received prior systemic treatment, and 23.6% to 44% had been treated with biologics, with over one-third failing prior biologic therapy.

Both secukinumab doses (150 mg and 300 mg) outperformed placebo in the primary efficacy endpoints, achieving PASI 75 responses (69.2% and 79.4%, respectively, vs. 4.2% for placebo) and IGA 0/1 responses at week 12 (51.4% and 65%, respectively, vs. 2.2% for placebo). Similarly, both doses were significantly more effective than etanercept. Consistent results were observed across subgroups and studies. Expectedly, patients previously exposed to systemic therapies showed slightly lower response rates than systemic-naïve patients. For instance, in study A2302, the IGA 0/1 response at week 12 for secukinumab 300 mg was 63.2% in pre-exposed patients versus 69.5% in naïve patients, with corresponding PASI 75 rates of 77.9% and 89.0%, respectively [4,5,6,7,8,9,10].

Secondary endpoints, including quality-of-life measures, corroborated the primary efficacy analysis. Response rates for PASI 90, PASI 100, and IGA were significantly higher in both secukinumab groups, with consistently superior outcomes observed with the 300 mg dose. Notably, the median time to achieve a 50% reduction in baseline PASI occurred at week 3 with secukinumab 300 mg. A plateau effect was observed at week 16, followed by a slight decline. Long-term maintenance data indicated that the effects at week 12 were reasonably sustained through week 52, particularly with the higher dose. The probability of response loss at week 52 was 12.9% with 300 mg and 24.7% with 150 mg. Relapse rates were also lower (7.4%) with the higher dose. Flexible maintenance regimens, as evaluated in study A2304, failed to demonstrate non-inferiority compared to fixed monthly dosing for maintaining long-term efficacy, leading the CHMP to recommend the 300 mg dose for all patients with continuous administration.

The safety profile of secukinumab aligns with that of other biologics used in psoriasis treatment [5,6,7,8], primarily involving infections (28.7% for secukinumab vs. 18.9% for placebo). Rare adverse events included neutropenia (0.3%) and hypersensitivity reactions (0.6%), which were mostly mild. No increased incidence of mycobacterial or serious opportunistic infections has been reported. Secukinumab slightly increased the risk of mucocutaneous Candida infections, herpes simplex infections, and upper respiratory tract infections compared to placebo and etanercept, all of which were mild and manageable without treatment discontinuation. Other reported side effects included conjunctivitis and gastrointestinal symptoms, predominantly diarrhea. Injection site erythema occurred less frequently (0.1%) than with etanercept (5%). The safety profile of the 300 mg dose was similar to that of the 150 mg dose. Although no association with cardiovascular events or malignancies has been observed, long-term follow-up data remain limited, necessitating further monitoring through post-marketing registries [9,10,11].

More than 150 publications pertaining to clinical trials or real-world studies provide clinical evidence of its efficacy in treating psoriasis as well as other conditions [12].

Secukinumab was authorized in Spain in 2015 to treat moderate-to-severe plaque psoriasis in adults who were deemed suitable for systemic therapy [13,14]. It is also currently approved for the treatment of moderate-to-severe plaque psoriasis in children and adolescents aged 6 years and older, psoriatic arthritis (PsA) in adults, enthesitis-related arthritis in the juvenile population, radiographic and non-radiographic axial spondyloarthritis, and suppurative hidradenitis [13,14].

Since its approval, numerous studies have been published on the real-world use of secukinumab, confirming its effectiveness and safety in treating patients with moderate-to-severe psoriasis. This article reviews real-world studies that were carried out in Spain to examine the efficacy and/or survival, in addition to the safety, of secukinumab in treating moderate-to-severe plaque psoriasis.

## 2. Materials and Methods

On 1 December 2022, a literature search was conducted on PubMed, Embase, and the Cochrane Library using the following terms: ((secukinumab) AND (psoriasis)) AND (Spain), including studies published in the last 5 years in English or Spanish, excluding conference abstracts. This cut-off point was established since there have been no further real clinical practice series since then.

Publications that provided data on the effectiveness and/or survival of secukinumab for the treatment of plaque psoriasis in actual clinical practice in Spain were considered. Studies focused on safety or quality-of-life analyses, pharmacoeconomic studies, clinical trials, meta-analyses, clinical practice guidelines, consensus, studies with fewer than ten patients, or series updated in subsequent publications were excluded (Table 1).

Each study was analyzed to determine its type, population evaluated, performance location and period, primary objectives, and variables. The following information was collected: (1) sample size; (2) clinical and baseline demographic characteristics of patients; (3) assessment of quality of life using the Dermatology Life Quality Index (DLQI); (4) assessment of severity using the Psoriasis Area Severity Index (PASI) and Body Surface Area (BSA); (5) treatment survival rate (percentage of patients who remain on treatment during follow-up) or estimate of the probability of survival and reason(s) for stopping treatment, including discontinuation due to adverse events (AEs).

## 3. Results

### 3.1. Literature Review

**Figure 1:**
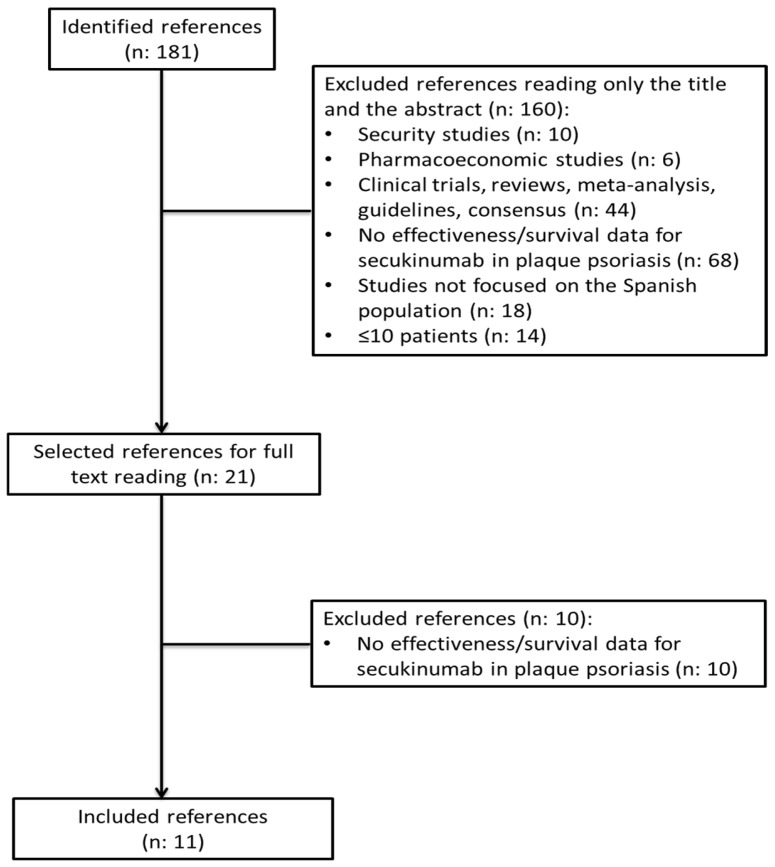
Flowchart of identified and included references.

We identified 181 references and eventually included 11 in this review (Figure 1).

Of the 11 references included, three studies by Ruiz-Villaverde et al. have finally been synthesized. The last one published with 171 patients, since the first series published by this group includes effectiveness data in 100 patients, the second and third are unique references and provide long-term efficacy and overall survival data for 171 patients [15] and for patients with or without PsA. Regarding the series by Daudén et al. [16], all studies from the same population group that provide survival and effectiveness data have been synthesized.

Supplementary Material Table S1 shows the main characteristics of the references included [15,16,17,18,19,20,21]. They included a total of 1050 patients in seven studies: six retrospective and one prospective [19].

### 3.2. Baseline Patient Characteristics

Table 2 summarizes the clinical and demographic characteristics of the patients [15,16,17,18,19,20,21]. The mean age ranged from 46.5 (standard deviation [SD]: 12) to 53.0 (DE: 14.7) years. The proportion of women was 28–43%, except in one study, where it was 55%. The body mass index (BMI) was ≥30 kg/m^2^ in 42.6–53.1% of the patients. The percentage of patients with PsA was 30–37%, except for one study, in which it was 53.1%. Of note, 27.9–35.9% of the patients were naïve to treatment with biologics, except in one study, in which the proportion was 47.4%. The mean baseline PASI score ranged from 12.5 (SD: 6.9) to 18.1 (SD: 10.3).

### 3.3. Secukinumab Dosage

Maintenance treatment with secukinumab was performed according to the label, with a dose of 300 mg in 857 patients (81.6%). A different dosage (in terms of induction, spacing, or at a dose of 150 mg) was followed in 115 (10.9%) of the total number of patients in two studies. The dose was not specified for 78 patients (7.4%). In Spain there is no national regulation that indicates whether the dermatologist should use the dose specified in the technical data sheet to start or continue the biological treatment and the data may vary between different autonomous communities and even between different hospitals in the same community.

### 3.4. PASI Severity Assessment

**Figure 2:**
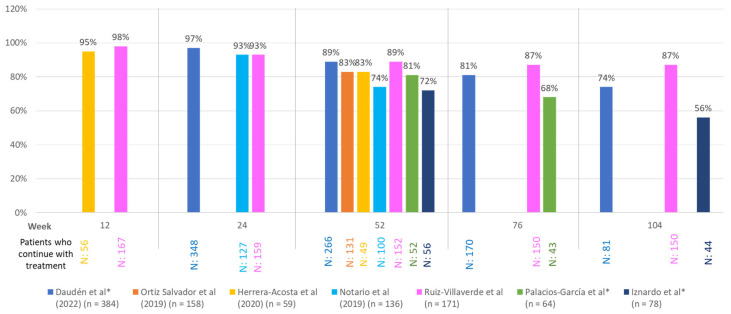
Survival outcomes of secukinumab in real-world practice studies [15,16,17,18,19,20,21]. Survival rates are shown, except for Iznardo et al. [21], who show an estimate of the probability of survival. * The number of patients still under follow-up has been calculated from the data.

Five references (n = 731) evaluated the percentage of patients who attained PASI 75 and PASI 90 in the short and long term (Figure 2 and Supplementary Material Figure S1).

The percentage of patients with PASI 90 response was 54–74% at Week 24 and 46–63% at Week 52.

A reference was identified that included data on PASI 100 (n = 59), being 42% at both Weeks 12 and 52. We identified two studies and three references describing the proportion of patients with absolute PASI ≤ 1. The proportion of patients with PASI ≤ 1 ranged from 43–47% at Week 12 to 47–56% at Week 52. Two referrals included the mean PASI at Weeks 4, 12, 16, 24, and 52, with a total of 294 patients, and one reference included the median PASI.

### 3.5. Survival with Secukinumab

Eight references and seven studies with secukinumab survival data were found (n = 1050 patients). Overall survival at Week 52 was 72–89%. The multicenter study by Ruiz-Villaverde et al. (n = 171) assessed long-term survival and observed a treatment survival rate of 87.0% (149/171 patients) maintained from Week 76 to Week 132 (Figure 2). In the largest series analyzed (Daudén et al., n = 384) [16], survival at Week 52 was 89.0%, and survival at Week 104 was 74.3%.

Table 3 shows the details of the patients who discontinued treatment, the reasons for the same, and the times at which discontinuation occurred. In the broader series (Daudén et al. [16], 83 of 384 patients (21.6%) discontinued treatment over 2 years of follow-up: 16.7% due to inefficacy and 3.4% due to AEs.

The primary causes of treatment discontinuation with secukinumab in patients with moderate-to-severe psoriasis were related to infectious events, as reported in multiple studies. Notably, no cases of discontinuation were associated with neutropenia, candidiasis, or the onset or reactivation of inflammatory bowel disease. According to Herrera et al. [17], adverse events leading to treatment suspension included erythematous toxicoderma, dyspnea during medication administration, and recurrent respiratory infections, affecting 5% of patients in the secukinumab group. Iznardo et al. reported one case of treatment discontinuation due to infectious ecthyma [21], while Notario et al. [18] described serious adverse events leading to withdrawal in two patients: one with a lower respiratory tract infection and another with reactivation of systemic sarcoidosis. In contrast, Ortiz et al. [19] found no cases of suspension due to adverse events. Palacios-García et al. noted a 6.7% discontinuation rate due to adverse events, although specific causes were not detailed, apart from 1.7% related to cancer diagnoses [20]. Ruiz-Villaverde [15] highlighted infectious causes as a key reason for treatment discontinuation, underscoring the relevance of monitoring infections in patients receiving secukinumab.

## 4. Discussion

In this review, we analyzed data on the effectiveness and survival associated with secukinumab treatment in patients with moderate-to-severe psoriasis in real-world studies in Spain. The results showed that secukinumab was effective and safe, induced an early response, and that most patients remained on long-term treatment.

The baseline characteristics of the patients in the included studies were generally comparable, although they differed slightly from those of patients included in phase III clinical trials such as ERASURE and FIXTURE, SCULPTURE, or CLEAR [4,5,6,7,8,9,10,11]. In the series analyzed, most patients had already tried biologics, and there was a higher proportion of patients with psoriasis and concomitant PsA, in contrast to clinical trials of secukinumab in psoriasis, in which patients were predominantly naïve to biologics and had lesser concomitant PsA. On the other hand, patients in clinical trials had a slightly higher mean baseline PASI than reflected in our review (22–25 vs. 12–18, respectively) [4,5,6,7,8,9,10,11].

Despite baseline differences, the clinical response rates (PASI 75 and PASI 90) in larger studies are consistent with the responses observed in phase 3 clinical trials. The PASI 75 response at Week 52 ranged from 64% in the smallest series (Herrera-Acosta et al., n = 59) [17] to 79% in the series by Ortiz-Salvador et al. (n = 158); the latter included patients from 12 hospitals and was the only prospective study [19].

In the CLEAR study, which compared secukinumab with ustekinumab, the proportion of patients who achieved a PASI 75 response at Week 52 was 92.5% higher than that noted in this review. However, baseline characteristics in the CLEAR study differ from the real-life series, primarily in the ratio of naïve to biological patients. Prior use of biologics was a key factor that decreased the efficacy of successive biologic treatments [22,23,24,25]. In addition, the use of two or more prior biologics with different mechanisms of action increased the likelihood of treatment discontinuation with secukinumab [16]. About 86% of patients in the secukinumab arm in the CLEAR study were naïve to biologics, while in the studies reviewed, this proportion was three times lower (≈30%). This could explain, at least in part, the differences in efficacy between the CLEAR study and the effectiveness of real-life studies, as well as the inclusion of an active control comparison arm (ustekinumab).

Regarding the PASI 90 response, the real-life study with more patients and prospectives (Ortiz-Salvador et al.) showed the highest response rate at Week 52 (63%) [19]. This response rate appears to be lower than that of the CLEAR study (76.2%) [22], but is in line with the response obtained in other phase III studies such as SCULPTURE [10] or ERASURE [9] (59.7% and 60.0%, respectively) and also with a meta-analysis by Augustin et al. that included 43 real-life studies with secukinumab, in which a PASI 90 response rate of 60% at 12 months was observed [26].

Two of the studies analyzed provide response rates with an absolute PASI between 0 and 1. In both cases, the proportion of patients with PASI ≤ 1 was close to 50% as of Week 24 [16,18]. These data are consistent with those of the phase III MATURE trial (the largest of the phase 3 clinical trials published to date), in which the PASI 100 rate at 52 weeks was 55.4% [27]. This study examined the efficacy and safety of the 300 mg/2 mL self-injector of secukinumab (launched in 2021) and demonstrated similar efficacy and safety to 2 self-injectors of the 150-mg dose [27], which was the dose used in the studies included in this review.

It is worth recalling that a favorable evolution of the PASI response with secukinumab has been associated with a parallel improvement in disease-related quality of life [28]. In a sub-study by Chicharro and Daudén et al. [16,28], an improvement in the quality of life associated with the PASI response measured by DLQI and other scales such as the Patient’s Global Psoriasis Assessment, Itch Numerical Rating Scale, or the EuroQoL Thermometer Visual Analog Scale was observed. These rates improved rapidly in the first 3 months of treatment and then reached a plateau that was maintained later [28].

Regarding survival, the results reported in real-life are consistent between studies and phase 3 trials [9,10]. The British Association of Dermatologists Biologics and Immunomodulators Register (BADBIR) compared the survival of adalimumab, ustekinumab, and secukinumab and observed secukinumab survival rates of 88% and 77% at 12 and 24 months, respectively, like the study by Daudén et al. (n = 384), in which the cumulative survival rates of secukinumab were 89.0% and 74.3% at 12 and 24 months, respectively [16]. These survival rates are like those described in the meta-analysis by Augustin et al., which included 43 real-life studies with secukinumab (90% and 80% at 6 and 12 months, respectively) [26]. Similarly, in a European study of 1756 patients with psoriasis, retention rates of secukinumab treatment after 1, 2, and 3 years in the study were 88.0%, 76.4%, and 60.5%, respectively [29].

Galluzo et al. [30] evaluated the long-term persistence, effectiveness, and safety of secukinumab in treating moderate-to-severe plaque psoriasis over six years across eight Italian dermatology centers. A total of 166 adult patients were included, with follow-ups conducted from treatment initiation between October 2015 and June 2017 up to 288 weeks. Secukinumab demonstrated sustained effectiveness, as the mean baseline PASI score of 18.1 significantly decreased to 0.7 after six years. The persistence rate was 86.8% at 24 months and 66.4% at 72 months, with primary reasons for discontinuation including loss of efficacy (58.6%) and adverse events (17.1%). Key predictors of discontinuation were genital psoriasis (hazard ratio [HR] = 2.30) and obesity (HR = 1.74). The safety profile remained consistent with known data, with adverse events including mucocutaneous fungal infections (3%), cardiovascular disturbances (15.2%), and metabolic alterations.

On the other hand, Gargiulo et al.’s retrospective multicenter study [31] analyzed the long-term persistence (“drug survival”) of interleukin (IL) inhibitors, specifically IL-12/23, IL-17, and IL-23, in 5932 treatment courses for moderate-to-severe plaque psoriasis. The findings revealed significant variability in drug survival across therapies, with IL-23 inhibitors generally showing superior outcomes. Risankizumab, an IL-23 inhibitor, demonstrated the highest persistence rates, with 91.6% of patients remaining on treatment after four years. By contrast, secukinumab, an IL-17 inhibitor, had a survival probability of 74.7%, among the lowest recorded.

The primary reasons for drug discontinuation included primary ineffectiveness, loss of effectiveness over time, adverse events, and patient preference. Bio-naive patients—those with no prior exposure to biologics—had better survival rates than those switching from another treatment. The study further noted that patients with psoriatic arthritis or specific comorbidities did not exhibit statistically significant differences in persistence, underscoring the importance of individual drug response over systemic factors.

The study attributed the observed differences in survival to the higher efficacy and tolerability profiles of IL-23 inhibitors, supported by previous clinical and real-world studies. Variability also stemmed from the availability of newer drugs during the study period, which may have influenced switching behaviors

Although in this study we focused primarily on effectiveness and survival data, all series were consistent in terms of the good safety profile of the drug, without a high rate of discontinuation of treatment due to AEs [4,5,6,7,8,9,10,11]. In this sense, and in line with what has been observed in different real-life studies [26,29], it is worth noting that according to the BIOBADADERM registry [32], secukinumab was considered a safe drug in routine clinical practice in Spain.

The main limitations of this review are those of studies evaluating real-life studies, which are, by definition, open label and sometimes heterogeneous, small, retrospective, and without an active control. In our case, a few studies included a small number of patients from a single hospital, but there were also large multicenter series. On the other hand, the fact that many studies do not have data for all time points makes the estimates less powerful. However, the final number of patients evaluated is large, and there are values at different time points. Hence, we believe valid conclusions can be drawn that can be extrapolated to the general population in Spain. On the other hand, the arrival of new biological treatments such as ixekizumab, brodalumab, bimekizumab, guselkumab, risankizumab, or tildrakizumab may have had effects on the survival associated with secukinumab [33]. In addition, the series predates the publication of the 2022 update of the Spanish Guidelines for the Treatment of Psoriasis with Biological Therapies [34], Therefore, the management of patients and the objectives set may differ slightly from current standards. Additionally, the studies collected were conducted with the use of 2 150-mg devices and did not incorporate the benefits of the 300-mg auto-injectable launched in 2021. This presentation, as recently published, presents non-inferior and numerically superior efficacy results, improving comfort for the patient [27]. In addition, the data are reported with different types of analyses (by protocol, by intent to treat, and with non-response imputation); hence, they are not directly comparable. Information about the type of analysis is specified in the graphs for correct interpretation.

## 5. Conclusions

Secukinumab is used to treat moderate-to-severe plaque psoriasis in real-world clinical practice in Spain. This review highlights the consistent efficacy, safety, and treatment persistence of secukinumab in the real-world management of moderate-to-severe plaque psoriasis in Spain. The data analyzed reveal that secukinumab induces rapid and sustained clinical improvements, with PASI 90 response rates ranging between 46% and 63% at Week 52 and survival rates exceeding 70% at two years. These outcomes align with results from clinical trials, such as SCULPTURE and CLEAR, confirming the robustness of secukinumab’s therapeutic profile. Despite minor baseline discrepancies, including the higher prevalence of biologic-experienced patients in real-world settings, the treatment demonstrated consistent effectiveness across diverse patient populations. Furthermore, secukinumab’s safety profile remained favorable, with adverse event-related discontinuation rates being minimal, corroborating its role as a reliable option for long-term management. Limitations of this analysis include the inherent variability of real-world studies, such as retrospective designs, heterogeneity, and small sample sizes in some cases. However, the large cumulative patient cohort across the studies reviewed strengthens the generalizability of these findings to clinical practice. The emergence of newer biologics underscores the need for continued comparative studies, yet secukinumab remains a benchmark therapy for psoriasis treatment. Future research should focus on integrating updated guidelines, new drug delivery systems, and long-term safety data to optimize therapeutic strategies further. This comprehensive evaluation underscores the pivotal role of secukinumab in addressing the clinical and quality-of-life challenges faced by patients with moderate-to-severe psoriasis.

## Figures and Tables

**Table 1:** Inclusion and exclusion criteria applied in the research.

Inclusion Criteria	Exclusion Criteria
Plaque psoriasis Effectiveness and/or survival associated with secukinumab treatment Specific data on clinical practice in Spain Language: English and/or Spanish Last 5 years (1 December 2017–1 December 2022)	Safety studies Pharmacoeconomic studies Clinical trials, reviews, meta-analyses, clinical practice guidelines, consensus Studies without specific data on effectiveness or survival of secukinumab in plaque psoriasis Studies not focused on the Spanish population Isolated cases (≤10) Conference abstracts

**Table 2:** Baseline patient characteristics.

Study	n	Age (Years) Mean (SD)	Sex (Female) n (%)	Weight (kg) Mean (SD)	Height (cm) Mean (SD)	BMI (kg/m^2^) Average (DE)	BMI ≥ 30 (kg/m^2^) n (%)	Disease Duration (Years) Mean (SD)	PsA n (%)	Biologic-Naïve Patients n (%)	PASI Mean (SD)	DLQI Mean (SD)	BSA Mean (SD)
Daudén et al. (2021) [16]	384	47.6 (12.5)	143 (37.2%)				163 (42.6%)		115 (30.0%)	119 (31.0%)	14.3 (8.4)		
Herrera-Acosta et al. (2020) [17]	59	48 (12.7)	24 (40.7%)			31.6 (5.5)			20 (33.9%)	21 (35.6%)	18.1 (10.3)		
Notario et al. (2019) [18]	136	49 (12.7)	39 (28.7%)	88.0 (19)	170.3 (9.1)	30.1 (6.8)	61 (44.9%)	22.4 (10.8)	45 (33.1%)	38 (27.9%)	13.5 (7.8)		
Ortiz-Salvador et al. (2019) [19]	158	46.5 (12)	68 (43.0%)	86 (20.1)		30.8 (7)	71 (44.9%)		55 (34.8%)	52 (32.9%)	12.5 (6.9)	12.8 (3.9)	
Palacios-García et al. (2019) [20]	64	50.5 (11.8)	26 (40.6%)				34 (53.1%)		34 (53.1%)	23 (35.9%)			
Ruiz-Villaverde et al. (2021) [15]	171	47.1 (14.5)	94 (55.0%)	86 (20)	169.7 (7.7)			18.3 (11.5)	64 (37.4%)	55 (32.2%)	13.8 (7.7)	13.0 (5.7)	
Iznardo et al. (2021) [21]	78	53.0 ± 14.7	26 (33%)						24 (31%)	37 (47.4%)			

BMI, body mass index; BSA, body surface area; DLQI, dermatology life quality index; PASI, psoriasis area severity index; PsA, psoriatic arthritis; SD, standard deviation.

**Table 3:** Discontinuing patients and reasons for discontinuation at different time cut-off points.

Study *	n	Reason for Discontinuation **	Week 12 (n)	Week 16 (n)	Week 24 (n)	Week 52 (n)	Week 76 (n)	Week 78 (%)	Week 104 (n)	Total Discontinuing Patients n (%)
Dauden et al. (2021) [16]	384	Lack of effectiveness	6		11	18	20		11	83 (21.6%)
		AE	2		4	6	3			
		Patient decision	1 (Desire for pregnancy)				1 (Lack of adhesion)			
Notario et al. (2019) [18]	136	Lack or loss of response			6	23				36 (26.5%)
		AE		2						
		Loss of follow-up			1	1				
		Other causes				3				
Ortiz-Salvador et al. (2019) [19]	158	Lack of effectiveness				8				27 (17.1%)
		Loss of efficiency				15				
		Lack of follow-up				4				
Palacios-García et al. (2019) [20]	64	Inefficiency						23.6%		- (32%)
		AE						6.7%		
		Cancer diagnosis						1.7%		
Ruiz-Villaverde et al. (2021) [15]	171	Primary failure	3							22 (13%)
		Secondary failure			5	3	2			
		AE	1		1	1	1			
		Loss to follow-up			2	1				
		Improvement in symptoms				2				
Iznardo et al. (2021) [21]	78									42 (53.8%) ***
Herrera-Acosta et al. (2020) [17]	59									10 (16.9%) ****

AE, adverse event. * In all studies, discontinuation was defined as discontinuation of treatment for any reason (lack or loss of efficacy, AEs, loss of follow-up, or other). ** The translation expresses the categories as they appear in the respective articles. *** This study indicates the number of patients who discontinued treatment (37 due to lack of efficacy, 2 due to AE, and 3 due to other causes), but does not indicate at which time points they discontinued or the n (%) at each time point. **** In this study, there are no details of the reasons for discontinuation per week. Only 3 out of 59 patients (5%) discontinued due to AEs.

## Supplementary Materials

The following supporting information can be downloaded at: https://www.mdpi.com/article/10.3390/jcm14020478/s1, Figure S1: Effectiveness results (PASI 75); Table S1: Main characteristics of included studies.
