# Supplementary material for: Secukinumab for Treatment of Plaque Psoriasis in Real-World Clinical Practice in Spain: A Literature Review
Source: J Clin Med. 2025 Jan 13;14(2):478. doi: 10.3390/jcm14020478 (PMC11766143; doi:10.3390/jcm14020478)
Supplement: Supplementary file 1 [file jcm-14-00478-s001.zip › jcm-3402507-supplementary.pdf]

Supplementary Materials

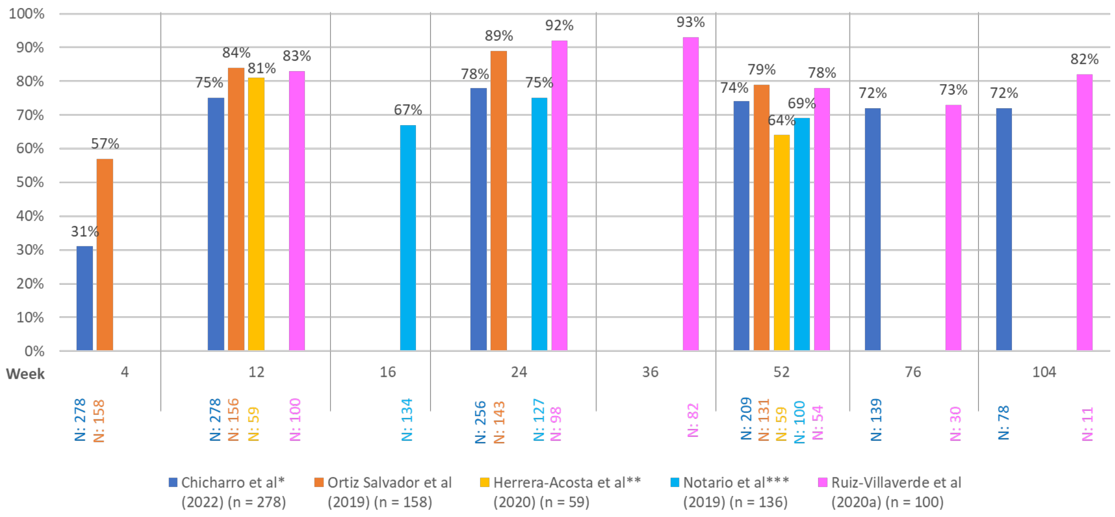

Figure S1. Effectiveness results (PASI 75). *Chicharro et al.: analysis of PASI 75 only in patients with PASI ≥10 at baseline. The intention-to-treat analysis, last observation considered is shown. **Herrera-Acosta et al.: analysis NRI (non-responder imputation). ***Notario et al.: analysis as treated. Other studies: type of analysis not specified.

Table S1. Main characteristics of included studies.

Reference	Document type	Design	Place	Follow-up	Objectives	Population	n*
Daudén et al. (2021) [16], Chicharro et al. (2022) [28]	Short and original report	Retrospective, multicenter	20 hospitals in Spain	104 weeks	Survival, safety, and effectiveness	Plaque psoriasis patients treated with secukinumab	384
Herrera-Acosta et al. (2020) [17]	Original	Retrospective, unicentric	Hospital Virgen de la Victoria, Málaga	52 weeks	Efficacy, safety, and survival of secukinumab and ixekizumab	Patients with moderate-to-severe psoriasis treated with secukinumab or ixekizumab	59
Notario et al. (2019) [18]	Original	Retrospective, multicenter	10 hospitals in Catalonia and Balearic Islands	52 weeks	Efficacy, safety, and survival	Patients with moderate-to-severe plaque psoriasis treated with secukinumab	136
Ortiz-Salvador et al. (2019) [19]	Original	Prospective, multicenter	12 hospitals in Spain	52 weeks	Effectiveness and survival	Patients with moderate-to-severe plaque psoriasis treated with secukinumab	158
Palacios-García et al. (2019) [20]	Letter	Retrospective, unicentric	Hospital Universitario Central de Asturias	76 weeks	Survival	Patients with psoriasis treated with secukinumab	64
Ruiz-Villaverde et al. (2021) [15]	Brief communication (2021) and original (2020b)	Retrospective, multicenter	5 hospitals in Andalusia	152 weeks	Survival	Patients with moderate-to-severe psoriasis treated with secukinumab	171
Iznardo et al. (2021) [21]	letter	Retrospective, unicentric	Hospital de la Santa Creu I Sant Pau (Barcelona)	71 weeks	Survival of guselkumab, ixekizumab, and secukinumab	Patients with moderate-to-severe psoriasis treated with guselkumab, ixekizumab, or secukinumab	78

*If there are more drugs in the study, the n of secukinumab is shown.
